# Serological and Molecular Study of the Duffy Blood Group among Malarial Endemic Region Residents in Brazil

**DOI:** 10.1590/0037-8682-0490-2021

**Published:** 2022-08-05

**Authors:** Dante Langhi, Sérgio Albuquerque, Rui Serafim, Gustavo de Carvalho Duarte, Dimas Tadeu Covas, José O. Bordin

**Affiliations:** 1 Universidade Federal de São Paulo, Departamento de Oncologia Clínica e Experimental, São Paulo, SP, Brasil.; 2 HHemo Hemoterapia SA, São Paulo, SP, Brasil.; 3 Fundação Hemocentro do Amazonas, Manaus, AM, Brasil.; 4 Universidade de São Paulo, Faculdade de Medicina de Ribeirão Preto, Ribeirão Preto, SP, Brasil.; 5 Universidade Estadual de Campinas, Centro de Hematologia e Hemoterapia, Campinas, SP, Brasil.

**Keywords:** Duffy blood group, Malaria, Blood donors, Genotype

## Abstract

**Background:**

The atypical chemokine receptor 1 (ACKR1) gene encodes the Duffy blood group antigens in two allelic forms: FY*A (FY*01) and FY*B (FY*02), which define the Fy(a+b-), Fy(a-b+), and Fy(a+b+) phenotypes. FY*BES (FY*02N.01) is a single T to C substitution at nucleotide -67 that prevents the FY*B from being expressed in red blood cells (RBCs).

**Methods:**

We evaluated 250 residents from a Brazilian malarial endemic region (RsMR). All individuals were phenotyped for Fy^a^ and Fy^b^ antigens and genotyped for FY*A, FY*B, FY*B ^*SE*^ , and FY*B ^*weak*^ alleles.

**Results:**

Among the 250 individuals, 209 (83.6%) reported previous malaria infection, and 41 (16.4%) did not. The Fy(a+b+) phenotype was present in 97/250 (38.8%), while the Fy(a-b-) was present in 7/250 (2.8%). The FY*A/FY*B was found in 130/250 (52%) and the FY*A/FY*A in 45/250 (18%). The c.1-67>TC was present, in homozygosity, in 11/250 (4.4%). Among 34 individuals with the Fy(a+b-) and FYA*/FYB* mutations, 4/34 (11.8%) had homozygosity for the c.1-67T>C. One individual presented the Fy(a+b-), FY*A/FY*B, and c.1-67T>C in homozygosis, whereas the other presented the Fy(a+b-), FY*A/FY*A, and c.1-67T>C in heterozygosis.

**Conclusions:**

We reported a low prevalence of the Fy(a-b-) in persons who had previously been infected with *Plasmodium vivax* (67.5%). We observed that 102/141 (72.3%) individuals expressing the Fy^b^ antigen had a *P. vivax* infection, indicating the importance of the Fy^b^ antigen, silenced by a c.1-67T>C mutation in homozygosis, in preventing the *P. vivax* infection. We showed that the c.1-67T>C mutation in the FY*A did not silence the FY*A expression on RBCs.

## INTRODUCTION

Malaria is a serious public health issue in Brazil, with roughly 145,000 cases recorded in 2014. The majority of occurrences occur in the Brazilian Amazonia (Amazon biome), a malaria-endemic region^1^.

However, recent migrations from the Amazonian region or other nations to non-Amazonian regions have resulted in secondary imported epidemics (i.e., introduced malaria)^2^.

*Plasmodium vivax*, *Plasmodium falciparum*, and *Plasmodium malaria* are the three principal *Plasmodium* species linked to native human malaria cases in Brazil^3^. 

The atypical chemokine receptor 1 (ACKR1), formerly known as Duffy antigen receptor for chemokines (DARC), is a minor blood group antigen that functions as both a chemokine receptor and a receptor for the malaria parasite *P. vivax* ^4^. Four alleles, five phenotypes, and five antigens comprise the Duffy system. Fy^a^ and Fy^b^ are antithetical antigens encoded by the co-dominant FY*A (FY*01) and FY*B (FY*02) genes. The minimal nucleotide polymorphism c.125G>A (rs12075) distinguishes these variants^5^. The FY*A allele's base is guanine (G), while the FY*B allele's base is adenine (A). This missense mutation results in the addition of a glycine codon to the FY*A allele and an aspartic acid codon to the FY*B allele at position 42 of the main product (p.Gly42Asp), defining the Fy(a+b-), Fy(a-b+), and Fy(a+b+) phenotypes^6^.

FY*X is a recessive allele of the FY*B (FY*BWK) gene located at the Duffy locus. The gene does not encode the synthesis of a Duffy system-specific antigen. The Fy^bwk^, also known as the Fy^x^ antigen, functions as a low-expression Fy^b^ antigen, and there is no known anti-Fy^bwk^ ^7^. The Fy^bwk^ phenotype is caused by a single missense mutation in the FY*B gene's coding area, c.265C>T (rs34599082), which results in the amino acid change p.Arg89Cys in gp-Fy^8^. Another mutation, c.298G>A (rs13962), has been found, resulting in the amino acid change p.Ala100Thr. Both variants are present in the FY*B and FY*A alleles^9^. The amino acid alteration occurs in the gp-first Fy's intracellular loop, resulting in ACKR18's relatively low membrane expression. Weak serological reactivity of the Fy^a^ antigen has already been found when the FY*A allele expresses the two mutations 265T and 298A.

The anti-Fy^a^ and anti-Fy^b^ antibodies among Caucasians define the Fy(a+b-), Fy(a+b+), and Fy(a-b+) phenotypes that most of the time represent the genotypes FY*A/FY*A, FY*A/FY*B, and FY*B/FY*B, respectively^10^. 

The null phenotype Fy(a-b-), also referred to as "erythrocyte silent" (ES; FyES), has been described more commonly in Afro-Americans and Occidental Africans but is uncommon in Caucasians^11^. Individuals with this phenotype are referred to as "Duffy-negative individuals," "ACKR1-null phenotype," "ACKR-1 null allele (FY-)," "Fy null allele," or "the null ES phenotype."^12^ The Fy(a-b-) phenotype is caused by homozygosity for the FY*B allele, which has the 5′ untranslated region point mutation c.1-67T>C (rs2814778). This mutation results in the formation of the FY*BES (FY*02N.01). Individuals with the Fy(a-b-) phenotype are homozygous for the c.1-67T>C polymorphism (C/C), which results in the absence of ACKR1 expression on FY- red blood cells (RBCs), whereas the heterozygous (T/C) and wild-type (T/T) states enable the expression of the Duffy molecule on FY+ RBCs 14. By inhibiting the binding location of the GATA-1 erythroid transcription factor, the C/C affects the ACKR1 promoter activity on RBCs^13^. The identical mutation has been identified on the FY*AES allele (FY*01N.01) in Papua New Guinea and Sudan residents, although only in the heterozygous state^14^ ^,^ ^15^. Pisacka et al. identified a new mutation in the FY* promoter region at position c.1-69 that likewise disrupts the GATA motif and results in the silence of the FY*A allele, resulting in the Fy null phenotype^16^.

The ACKR1 protein is required for the attachment of *P. vivax* merozoites and functions as bait and scavengers for chemostatic agonists^17^. The ACKR1-null allele confers resistance to malaria infections induced by *P. vivax* or *P. falciparum* ^18^. The related protective benefits may account for the natural selection-driven propagation of the ACKR1-null FY- (C/C) polymorphism in malaria transmission zones such as Western, Central, and Southeastern Africa, where the prevalence rate approaches 100%^19^. Recent investigations reveal that *P. vivax* malaria can be discovered in Duffy-positive and-negative or those whose DARC status is unknown^20^ ^-^ ^27^.

These data support the notion that this parasite is quickly developing, capable of invading erythrocytes via receptors different from those found in Duffy, which might significantly influence the existing distribution of *P. vivax* ^23^.

We investigated blood samples from the risk-standardized mortality rate (RsMR) in Brazil in this study. All participants were phenotyped for the Fy^a^ and Fy^b^ antigens and genotyped for the FY*A, FY*B, FY*BSE, and FY*B^weak^ alleles, and then the promoter and codifying regions of the FY gene were sequenced. Additionally, we associated the phenotypes and genotypes of the RsMR with prior *P. vivax* infection.

## METHODS

### Study population

The study population consisted of 250 RsMR individuals in the city Presidente Figueiredo, located in the Amazon State, Brazil. This Amazonian city has an annual parasite index (API) of 301.65 malaria cases per 1,000 people, making it a malaria-risk region^28^. All 250 individuals were interviewed regarding their previous status of malaria infection. They were all phenotyped for the Fy^a^ and Fy^b^ antigens, genotyped for the FY*A, FY*B, FY*ES, and FY*B^weak^ alleles, and then sequenced for the FY* promoter and coding areas. The study was authorized by the ethical committee of the Universidade Federal de São Paulo (UNIFESP).

### Phenotyping and genotyping studies

All blood samples were phenotyped using the gel agglutination technique (DiaMed-Latino América S.A, Lagoa Santa, MG, Brasil) and genotyped for the FY* alleles using the polymerase chain reaction-restriction fragment length polymorphism (PCR-RFLP) technique^29^. The promoter and coding regions of the FY* were sequenced using the ABI Prism®Big Dye™ Terminator Cycle Sequencing Ready Reaction Kit (Perkin Elmer). 

### Statistical analysis

The statistical analysis was performed using Pearson’s chi-square test (x^2^). Statistical significance was defined as a *P*-value < 0.05.

## RESULTS

The immunohematological results showed a predominance of the Fy(a+b+) and FY*A/FY*B combination among the residents of a malarial endemic region (RsER) [97/250 (38%)], The Fy(a-b+) phenotype was found in 7/250 (2.8%) RsER (Table 1).

**TABLE 1:** Distribution of the Duffy blood group phenotype and genotype among residents of a malarial endemic region (RsER).

Phenotype	Genotype	N	%
Fy(a+b+)	FY*A/FY*B	97	38.8
Fy(a+b-)	FY*A/FY*A	44	17.6
Fy(a+b-)	FY*A/FY*B	34	13.6
Fy(a-b+)	FY*B/FY*B	68	27.2
Fy(a-b-)	FY*B/FY*B	7	2.8
Total		250	100

The comparison of phenotype and genotype combinations and their relation to previous infection by *P. vivax* were analyzed in 209 RsER. The frequency of the previous *P. vivax* infection was significantly different among the various phenotype and genotype combinations (*P* < 0.001) (Table 2). The Fy(a+b+)/FY*A/FY*B and Fy(a-b+)/FY*B/FY*B combinations were found more frequently among individuals who had had previous *P. vivax* infection compared with those participants who had not been infected [59/141 (41.8%) *vs*. 19/68 (27.9%) and 43/141 (30.5%) *vs*. 14/68 (20.6%), respectively; (*P* < 0.001)]. The Fy(a+b-)/FY*A/FY*A and Fy(a-b-)/FY*A/FY*B combinations were more frequent in individuals with no previous history of *P. vivax* infection [15/68 (22.1%) *vs*. 25/141 (17.7%) and 7/68 (10.3%) *vs*. 0/141 (0%), respectively; (*P* < 0.001)]. We did not observe participants with the Fy(a-b-)/FY*B/FY*B combination who had had previous *P. vivax* infection (Table 2). 

**TABLE 2:** *P. vivax* malaria infection among the RsER, according to Duffy phenotype and genotype combinations.

		***P. vivax* infection**					
		Yes		No		Total	
Phenotype	Genotype	N	%	N	%	N	%
Fy(a+b+)	FY*A/FY*B	59	41.8	19	27.9	78	73.3
Fy(a+b-)	FY*A/FY*A	25	17.7	15	22.1	40	19.1
Fy(a+b-)	FY*A/FY*B	14	9.9	13	19.1	27	12.9
Fy(a-b+)	FY*B/FY*B	43	30.5	14	20.6	57	27.3
Fy(a-b-)	FY*B/FY*B	0	0.0	7	10.3	7	3.3
**Total**		**141**	**100**	**68**	**100**	**209**	**100**

*P* < 0.001.

The frequency of the c.1-67T>C mutation in homozygosis at the promoter region of the FY* gene was found in 11/250 (4.4%). Overall, we found a frequency of the c.1-67T>C mutation among the 84/250 (33.6%) RsER individuals. On the other hand, the majority of the RsER [166/250 (66.4%)] did not present the c.1-67T>C mutation (Table 3).

**TABLE 3:** Frequency of the c.1-67T>C mutation of the Duffy blood group among RsER.

	RsER	
Allele	N	%
W/W	166	66.4
W/M	73	29.2
M/M	11	4.4
**Total**	**250**	**100**

**W:** Wild; **M:** Mutate.

We investigated the frequency of the c.1-67T>C mutation that causes the silencing of the Fy^b^ antigen expression on RBCs in individuals showing a discrepancy between the phenotype [Fy(a+b-)] and genotype (FY*A/FY*B). We found that 30/34 (88.2%) individuals with the Fy(a+b-) phenotype and FY*A/FY*B genotype presented the c.1-67T>C mutation in heterozygosis (W/M) and 4/34 (11.8%) in homozygosis (M/M) (Table 4). All 34 individuals were also investigated for the c.265C>T and c.298G>A mutations in the coding region of the FY* **,** responsible for the weak expression of Fy^b^ (Fy^bweak^), but none of them carried such mutations. Four of 34 (11.8%) individuals presenting the Fy(a+b-) phenotype, the presence of the c.1-67T>C mutation, polymorphism of the FY*A and FY*B alleles, and c.265C>T and c.298G>A mutations. The FY*A/FY*B genotype and c.1-67T>C mutation in homozygosis were analyzed by nucleotide sequencing of the promoter and codifying regions of the FY* gene. In all four participants, we confirmed by DNA sequencing the results previously found using the PCR-RLFP method, *i.e*., the presence of the c.1-67T>C mutation in homozygosis and not the absence of the c.265C>T and c.298G>A mutations. These individuals had the Fy^b^ antigen, but not the Fy^a^ antigen expression, silenced on their RBCs (Table 4). 

**TABLE 4:** Frequency of the c.1-67T>C mutation and phenotype and genotype discrepancy among RsER .

	Fy(a+b-) / FY*A/FY*B					
	No		Yes		Total	
Allele	N	%	N	%	N	%
W/W	166	76.9	0	0	166	66.4
W/M	43	19.9	30	88.2	73	29.2
M/M	7	3.2	4	11.8	11	4.0
**Total**	**216**	**100**	**34**	**100**	**250**	**100**

**W:** Wild; **M:** Mutate.

The correlation of the *P. vivax* infection and the presence of the c.1-67T>C mutation was investigated among the RsER, showing a discrepancy between the Fy(a+b-) phenotype and FY*A/FY*B genotype. The c.1-67T>C mutation not found (W/W) in 103/141 (73.0%) of previously *P. vivax-*infected RsER, compared with 38/68 (55.9%) of non-infected RsER. The presence of the c.1-67T>C mutation in heterozygosis (W/M) was detected in 14/141 (9.9%) and 10/68 (14.7%) of the previously infected and non-infected RsER, respectively. All RsER (n = 10) carrying the mutation in homozygosis (M/M) with no history of previous *P. vivax* infection (*P* < 0.001) (Table 5). 

**TABLE 5:** *P. vivax* malaria infection among RsER with Fy(a+b-) phenotype and FY*A/FY*B genotype, according to the c.-1-67T>C mutation.

***P. vivax* Malaria**							
		Yes			No		
		Fy(a+b-) / FY*A/FY*B			Fy(a+b-) / FY*A/FY*B		
c.-1-67T>C mutation		No	Yes	Total	No	Yes	Total
W/W	n	103 (100%)	0	103 (100%)	38 (100%)	0	38 (100%)
							
W/M	n	24 (63.2%)	14 (36.8%)	38 (100%)	10 (50%)	10 (50%)	20 (100%)
							
M/M	n	0	0	0	7 (70%)	3 (30%)	10 (100%)
							
**Total**	**n**	**127 (90.1%)**	**14 (9.9%)**	**141 (100%)**	**55 (80.9%)**	**13 (19.1%)**	**68 (100%)**

We observed that among 68 RsER with no history of previous *P. vivax* malaria infection, 13/68 (19.1%) showed the Fy(a+b-) phenotype and the FY*A/FY*B genotype silencing the Fy^b^ expression on their RBCs. In this group of individuals, 10/13 (76.9%) presented the c.1-67T>C mutation in heterozygosis W/M), and 3/13 (23.1%) presented the c.1-67T>C mutation in homozygosis (M/M) (Table 5). The other 55/68 (80.9%) non-infected RsER did not show such a phenotype/genotype combination. 

Among the 141/209 (67.5%) RsER with previous malaria infection by *P. vivax*, 14/141 (9.9%) showed the Fy(a+b-) phenotype and FY*A/FY*B genotype combination, together with the c.1-67T>C mutation in heterozygosis (W/M), while 127/141 (90.1%) did not present the Fy(a+b-) phenotype and FY*A/FY*B genotype combination (Table 4). We observed that 14/38 (36.8%) individuals presented the Fy(a+b-) phenotype, FY*A/FY*B genotype, and c.1-67T>C mutation in heterozygosis (W/M) with previous *P. vivax* infection, but 10/20 (50%) had not been infected, suggesting that the c.1-67T>C mutation in heterozygosis did not provide full protection against the *P. vivax* infection (Table 5). As described above, 3/10 (30%) with the Fy(a+b-) phenotype, FY*A/FY*B genotype, and c.1-67T>C mutation in homozygosis (M/M) had not been infected.

The molecular studies analysis on this group of RsER allowed us to identify not only individuals presenting the Fy(a+b-) phenotype, FY*A/FY*B genotype, and c.1-67T>C mutation in homozygosis (M/M) but also one additional participant presenting the Fy(a+b-) phenotype, FY*A/FY*A genotype, and c.1-67T>C mutation in heterozygosis (W/M). However, we could not confirm the silencing of the Fy^a^ antigen because the individual had the FY*A allele in homozygosis, and the phenotyping showed the presence of the Fy^a^ antigen. This could be due to one FY*A allele not carrying the c.1-67T>C mutation, while the other FY*A allele presented the c.1-67T>C mutation; therefore, the silencing had not occurred. On the other hand, the c.1-67T>C presence in the FY*A did not silence the Fy^a^ antigen on RBCs.

## DISCUSSION

The Duffy antigens are important in the *P. vivax* malarial infection and as receptors for chemokines^30^ ^,^ ^31^. It is known that the Duffy-negative phenotype is predominant among Africans and Afro-American individuals^11^ ^,^ ^31^. The preponderance of the Fy(a-b-) phenotype among Africans is consistent with the genetic adaptation theory, as approximately 95% of individuals in Western Africa have the Duffy-negative phenotype, while *P. vivax* infection has virtually gone in those regions^32^. In contrast, in Southeast Asia, considered a highly endemic *P. vivax* area, the prevalence of the Fy(a-b-) phenotype is uncommon^33^. 

Brazil has a large malarial endemic area represented mainly by the Amazon region^28^, and Brazil has a highly mixed ethnic population. Such aspects offer a unique opportunity to study the variations of the Duffy phenotypes and genotypes and their possible associations with the *P. vivax* infection among populations living inside or outside the malarial endemic regions. As a result, we examined the Duffy phenotype and genotype distribution in one group of RsER in the current investigation (Amazon State, Northwestern Brazil). Additionally, we analyzed the association between Duffy phenotypes and genotypes with the *P. vivax* infection frequency in the group of RsER. 

We found a high frequency of the Fy(a+b+) phenotype among RsERs similar to that already reported in Caucasians but different from that detected in Asian and African populations^34^. The high rate of *P. vivax* malaria infection among RsERs suggests that, to some extent, a genetic selection mechanism against the parasite infection did not occur in the Amazon region, as compared with what happened in Western Africa^32^. 

Cavasini et al. found that the FYA/FYB genotype was the most prevalent among patients with malaria vivax in four parts of the Brazilian Amazon region, supporting the concept that persons with this genotype had a higher vulnerability to malaria^27^.

In our study, the frequency rate of previous *P. vivax* infection among RsER was not different for the FY*A/FY*A, FY*A/FY*B, and FY*B/FY*B genotypes distribution. Nevertheless, we observed that 102/141 (72.3%) of the individuals expressing the Fy^b^ antigen had a previously acquired *P. vivax* infection, indicating the biological importance of the Fy^b^ antigen, silenced by the c.1-67T>C mutation in homozygosis in preventing the *P. vivax* infection. 

The lower prevalence of Fy(a-b-) phenotype in the RsMR than that described among the African population probably reflects Brazilian miscegenation with people of African descent^11^. 

The Fy(a-b-) phenotype is almost undetectable in a malaria-endemic region of Southeast Asia, and the genetic foundation for this phenotype is distinct from that described in African adults with FY*BSE allele homozygosis^35^. Shimizu and colleagues have reported that in some Asian regions, people showing the Fy(a-b-) phenotype present the FY*A/FY*A and FY*A/FY*B genotypes but not the FY*B ^*SE*^ /FY*B ^*SE*^ genotype. The investigators also described the presence of the Fy^aweak^ antigen among individuals from Southeast Asia as an antigen weakly reacting with the anti-Fy^a^ sera; however, in the present study we did not find an analogous immunohematological variation. 

We found discrepancies between phenotypes and genotypes in 13.6% of the RsMR, including individuals showing the Fy(a+b-) phenotype but not the FY*A/FY*B genotype, resulting in the suppression of Fyb antigen expression on RBCs. These blood samples were investigated for the presence of the c.1-67T>C mutation in the promoter region of the FY* gene that characterizes the FY*B ^*SE*^ allele and the c.265C>T and c.298G>A mutations in the coding region of the FY* gene that characterize the FY*B ^*WEAK*^ allele. The Fy(a+b-) phenotype was present among 88.2% of the RsMR, showing the FY*A/FY*B genotype and the c.1-67T>C mutation in heterozygosis that silenced the expression of the Fy^b^ antigen on their RBCs. No individuals presented the c.265C>T or c.298G>A mutations. However, 12% of the RsER showed the FY*B ^*SE*^ allele in heterozygosis that corresponds to the most frequent allele found among Africans and Afro-Americans^12^. 

Individuals showing the c.1-67T>C mutation in heterozygosis in the FY*B allele (W/M) presented with the dose-effect regarding the expression of the Duffy antigen on RBCs; therefore, expressing only 50% of their antigens^9^. This condition suggests that heterozygosis for the c.1-67T>C mutation favors the protection against the *P. vivax* infection^36^. 

When we analyzed this group of RsER, according to the *P. vivax* infection and Duffy phenotype and genotype discrepancy, we observed that among 27 individuals presenting the Fy(a+b-) phenotype and the FY*A/FY*B genotype, 13 (48.2%) did not have an infection, while 14 (51.8%) had had an infection. Therefore, the infection rate was similar between the two groups, suggesting a tendency for a phenotype/genotype protection against the *P. vivax* infection, even though the numbers of individuals tested with such phenotype/genotype discrepancy were relatively small. However, when we included the analysis of the c.1-67T>C mutation status, characterizing the FY*A/FY*B ^*SE*^ genotype, the tendency for protection against the *P. vivax* infection was not confirmed because only 3 of the 13 individuals showing the Fy(a+b-) phenotype, the FY*A/FY*B genotype and no *P. vivax* infection presented the c.1-67T>C mutation in homozygosis.

Differently from our data, in which 100% (7/7) of the individuals presenting the FY*B ^*SE*^ /FY*B ^*SE*^ had no previous malaria infection, Carvalho *et al*. evaluated the resistance profile for *P. vivax* infection in Anajas, State of Pará, Brazil, among Duffy-negative and-positive individuals, found no significant difference between the two groups^26^. 

Zimmerman in New Guinea and Kempinska-Podhorodecka in Sudan described individuals showing the c.1-67T>C mutation in heterozygosis for the FY*A allele that silenced the Fy^a^ antigen expression on RBCs^14^ ^,^ ^15^. Additionally, Pisacka and coworkers described a Caucasian family with a T>C substitution at position c.1-69 in the FY* promoter region, which breaks the GATA motif (TTATCT>TCATCT) and is associated with the suppression of FY*A expression1. In our investigation, we discovered that 4/34 (11.8 percent) of participants with the Fy(a+b-) phenotype and the FY*A/FY*B genotype (n = 34) had the c.1-67T>C mutation in homozygosis, suggesting that the c.1-67T>C mutation in the FY*A allele did not suppress Fy^a^ antigen production on their RBCs. We also found one individual who presented the Fy(a+b-) phenotype, FY*A/FY*A genotype, and c.1-67T>C mutation in heterozygosis. Based on the phenotype analysis, we could not confirm whether the c.1-67T>C mutation in this individual silenced the Fy^a^ expression or not because we were not able to demonstrate if the Fy^a^ presence was in a duplicated form, corresponding to two alleles (FY*A/FY*A), or if there was one antigen silencing corresponding to the allele carrying the c.1-67T>C mutation. 

Two of the four patients presented no prior history of malaria infection; one had *P. falciparum* infection and one did not disclose the malaria epidemiology status. Individuals with the Fy(a+b-) phenotype, FY*A/FY*A genotype, and c.1-67T>C mutation in heterozygosis were infected with malaria by *P. vivax* and *P. falciparum.* Despite the small number of cases in the current data, the presence of the c.1-67T>C mutation in FY*A and FY*B alleles may confer some protection against *P. vivax* infection, given the low expression of the Duffy antigens on RBCs in individuals carrying the c.1-67T>C mutation, a situation that may be exacerbated by the presence of a double mutation. Additional research with people carrying the c.1-67T>C FY*A allele are required to substantiate our results that the Fy^a^ antigen is not silenced in their RBCs.
